# *Lactobacillus casei* Variety *rhamnosus* Probiotic Preventively Attenuates 5-Fluorouracil/Oxaliplatin-Induced Intestinal Injury in a Syngeneic Colorectal Cancer Model

**DOI:** 10.3389/fmicb.2018.00983

**Published:** 2018-05-15

**Authors:** Ching-Wei Chang, Chia-Yuan Liu, Hung-Chang Lee, Yen-Hua Huang, Li-Hui Li, Jen-Shiu Chiang Chiau, Tsang-En Wang, Cheng-Hsin Chu, Shou-Chuan Shih, Tung-Hu Tsai, Yu-Jen Chen

**Affiliations:** ^1^Institute of Traditional Medicine, National Yang-Ming University, Taipei, Taiwan; ^2^Division of Gastroenterology, Department of Internal Medicine, MacKay Memorial Hospital, Taipei, Taiwan; ^3^Department of Medical Research, MacKay Memorial Hospital, New Taipei City, Taiwan; ^4^Department of Medicine, MacKay Medical College, New Taipei City, Taiwan; ^5^Mackay Junior College of Medicine, Nursing, and Management, New Taipei City, Taiwan; ^6^MacKay Children’s Hospital, Taipei, Taiwan; ^7^Institute of Biomedical Informatics, Center for Systems and Synthetic Biology, National Yang-Ming University, Taipei, Taiwan; ^8^Department of Chemical Engineering, National United University, Miaoli, Taiwan; ^9^Department of Radiation Oncology, MacKay Memorial Hospital, Taipei, Taiwan

**Keywords:** FOLFOX, *Lactobacillus*, intestinal mucositis, apoptosis, gut microbiota

## Abstract

Adjuvant 5-fluorouracil (5-FU)-based chemotherapy, including FOLFOX (5-FU, leucovorin, and oxaliplatin), is recommended for colorectal cancer. However, intestinal mucositis remains a common adverse effect for which no effective preventive strategies are available. To develop a convenient and novel way to alleviate mucositis, we investigated the effect of *Lactobacillus casei* variety *rhamnosus* (*Lcr35*) on FOLFOX-induced mucosal injury. BALB/c mice subcutaneously injected with syngeneic CT26 colorectal adenocarcinoma cells were orally administered *Lcr35* daily before, during, and after 5-day injection of FOLFOX regimen, for 14 days. The following methods were used: diarrhea score for toxicity, ELISA for cytokine production, histopathology for intestinal injury, immunohistochemistry for apoptosis/proliferation and regulatory proteins, RT-PCR for cytokine mRNA expression, and DNA sequencing for fecal gut microbiota. FOLFOX administration to colorectal cancer-bearing mice significantly inhibited tumor growth and the accompanying marked diarrhea and intestinal injury histologically characterized by the shortening of villi and destruction of intestinal crypts. Preventive administration of *Lcr35* dose-dependently reduced the severity of diarrhea and intestinal mucositis without affecting the anti-tumor effect of FOLFOX. The numbers of apoptotic, NF-κB-, and BAX-activated cells increased after FOLFOX, and these responses were mitigated by *Lcr35*. TNF-α and IL-6 upregulation by FOLFOX treatment was attenuated by *Lcr35*. The fecal gut microbiota composition of *Firmicutes* and *Bacteroidetes* disturbed by FOLFOX was significantly reversed by *Lcr35* toward a preferential profile. In conclusion, the oral probiotic *Lcr35* prevented FOLFOX-induced intestinal mucositis in colorectal cancer-bearing mice. The putative mechanism might involve modulation of gut microbiota and proinflammatory responses with suppression of intrinsic apoptosis in intestinal injury.

## Introduction

Gastrointestinal toxicity due to chemotherapeutic drugs is a major cause of morbidity and mortality in cancer patients. Mucositis and diarrhea are the most significant enterotoxicities. Lesions associated with mucositis result in pain, decreased quality of life, increased length of hospitalization, higher risk of infection, and modification of anti-neoplastic treatment regimens (Sonis et al., 2004; Sharma et al., 2005; Lee et al., 2014). The pathophysiology of chemotherapy-induced mucositis remains unclear and involves a complex and dynamic array of biological events (Logan et al., 2007; Lee et al., 2014). Some studies have suggested a five-stage process, including an initiation phase, a message generation phase, a signaling and amplification phase, an ulceration phase, and a spontaneous healing phase (Sonis et al., 2004; Logan et al., 2007). The pathophysiology might include decreased villi length and disruption of crypt cell homeostasis, and several pathogenic elements are involved, including direct toxicity, a change in the balance of bowel microbial flora, oxidative stress, apoptosis, hypoproliferation, and abnormal inflammation. Recent studies have revealed that chemotherapeutics affect the intestinal microbial composition (Stringer et al., 2009a,b) and fecal microbiota (Touchefeu et al., 2014). However, no well-established or up-to-date therapeutic strategy is available to manage chemotherapy-induced intestinal mucositis (Sharma et al., 2005). Thus, the development of an effective intervention against chemotherapy-related mucositis is urgently needed for oncological supportive care.

Probiotics are live microorganisms that, similar to certain drugs or food supplements, help maintain a beneficial microbial balance in the digestive tract of humans and other hosts. Probiotics have been tested in multiple indications, including gastrointestinal disorders (for the prevention and treatment of infectious and antibiotic-induced diarrhea), treatment of liver insufficiency, lactose intolerance, inflammatory bowel disease, irritable bowel syndrome, and anti-tumorigenic activities (Liong, 2008; Jacouton et al., 2017). Certain strains of *Lactobacillus* have been recommended in the treatment and prevention of diarrhea and inflammatory bowel disease to improve the integrity of the intestinal tissue (Eizaguirre et al., 2002). Experimental and clinical evidence suggest that probiotics might have a beneficial effect on the toxicity of anticancer therapy (Mego et al., 2013). The most commonly used probiotic organisms are lactic acid bacteria, especially those belonging to the genus *Lactobacillus*. We previously demonstrated that *Lactobacillus* attenuates the barrier disruption of intestinal epithelial cells caused by *Salmonella* lipopolysaccharide administration and ameliorates chemotherapy-induced intestinal mucositis in a healthy mouse model (Yeung et al., 2013, 2015). Thus, probiotics, by manipulating gut microbiota, may ameliorate inflammation, and protect the epithelium by maintaining intestinal epithelial integrity and reduce the severity of mucositis.

Colorectal cancer is one of the most common types of cancer and a lethal disease worldwide (Brenner et al., 2014). For several decades, regimens based on 5-fluorouracil (5-FU), an anti-metabolite anticancer agent, have been the first-choice chemotherapy for colorectal cancer (Brenner et al., 2014). Among regimens based on 5-FU with the cytotoxic agent oxaliplatin, FOLFOX (5-fluorouracil, leucovorin, and oxaliplatin) has been widely used as standard chemotherapy for advanced and metastatic colorectal cancer (de Gramont et al., 2000; Giacchetti et al., 2000; Mohelnikova-Duchonova et al., 2014). While 5-FU alone has an objective response rate of ∼20%, the combination of oxaliplatin with 5-FU/folinic acid results in significantly increased response rates and improved survival (Mohelnikova-Duchonova et al., 2014). The 5-FU is a pyrimidine analog (Johnson and Rogers, 1964) that is transformed inside the cell into different cytotoxic metabolites, which are then incorporated into DNA and RNA, finally inducing cell cycle arrest and apoptosis. Oxaliplatin, a platinum derivative, functions by forming both inter- and intrastrand crosslinks in DNA to prevent DNA replication and transcription, resulting in apoptosis (Graham et al., 2004). Gastrointestinal toxicity is potentiated in the combination therapy of oxaliplatin with 5-FU in clinical studies; however, the underlying mechanism remains unclear (Kuebler et al., 2007; Bano et al., 2014; Lee et al., 2014).

Our previous researches have reported anti-inflammatory effects of the probiotic strain *Lactobacillus casei* variety *rhamnosus* (*Lcr35*) on lipopolysaccharide-induced inflammation and epithelial barrier dysfunction in a co-culture model using Caco-2/peripheral blood mononuclear cells (Fang et al., 2010). Furthermore, we recently demonstrated that this strain attenuates chemotherapy-induced intestinal mucositis in healthy mice (Yeung et al., 2015). The 5-FU-induced diarrhea and damage in jejunal villi was ameliorated following *Lcr35* administration. *Lcr35* suppressed the upregulation of pro-inflammatory cytokines expression in intestinal mucositis tissues following 5-FU treatment. No bacterial translocation was found in the safety study of *Lcr35* (Yeung et al., 2015). However, the beneficial role of *Lactobacillus casei* variety *rhamnosus* in FOLFOX-induced intestinal mucositis of colorectal cancer model remains to be assessed. Subcutaneously injected colorectal carcinoma murine models have been widely used in translational research (Liu et al., 2005; Doi et al., 2010; Kim et al., 2012; Evans et al., 2016; Gordon et al., 2017). In the current study, we further investigated the protective effect of *Lactobacillus casei* variety *rhamnosus* on intestinal mucosal injury induced by 5-FU-based chemotherapy (FOLFOX) in subcutaneously injected colon cancer mice. Our results revealed that probiotic *Lcr35* did not interfere anti-tumor effect of FOLFOX. *Lcr35* ameliorated diarrhea and repaired intestinal mucosa damage following FOLFOX treatment. The possible mechanism(s) of *Lcr35* was also elucidated.

## Materials and Methods

### Chemotherapy Regimen Administration

Chemotherapy regimen (FOLFOX) with 5-fluorouracil (5-FU, Sigma F6627), leucovorin (LV, Sigma F7878), and oxaliplatin (Sigma O9512) was injected intraperitoneally (i.p.) to cause mucositis and diarrhea. The drug-dosing schedule was based upon that used in previously published studies and our preliminary dose-finding experiments (El-Salhy et al., 2005; Wagner et al., 2009). The 5-FU and LV were injected i.p. at a single dose (30 and 10 mg/kg, respectively) for 5 consecutive days (days 0–4). On the first day (day 0), the experimental animals also received oxaliplatin (1 mg/kg, i.p.) 1 h after 5-FU/LV administration. Saline was injected i.p. in control groups.

### Probiotic Preparation

*Lactobacillus casei* variety *rhamnosus* (*Lcr35*) (Antibiophilus^®^) was diluted in sterile saline and administered by oral gavage. The mice received 100 μL of saline or suspension containing 1 × 10^3-7^ CFU of the probiotics cocktail daily during the experiment.

### Cell Culture

The CT26 cells, *N*-nitroso-*N*-methyl urethane-induced mouse colon carcinoma cells of BALB/c origin (Brattain et al., 1980), were purchased from the American Type Culture Collection. Cells were cultured in RPMI-1640 medium (Gibco) supplemented with 10% heat-inactivated fetal calf serum (Hyclone) at 37°C in a humidified incubator with 50 mL/L CO_2_. The cell cultures were passaged every 2–3 days with TEG solution (0.25% trypsin, 0.1% EDTA, and 0.05% glucose in Hanks’ balanced salt solution) and were maintained in exponential growth.

### Animal Experiments

**FIGURE 1:**
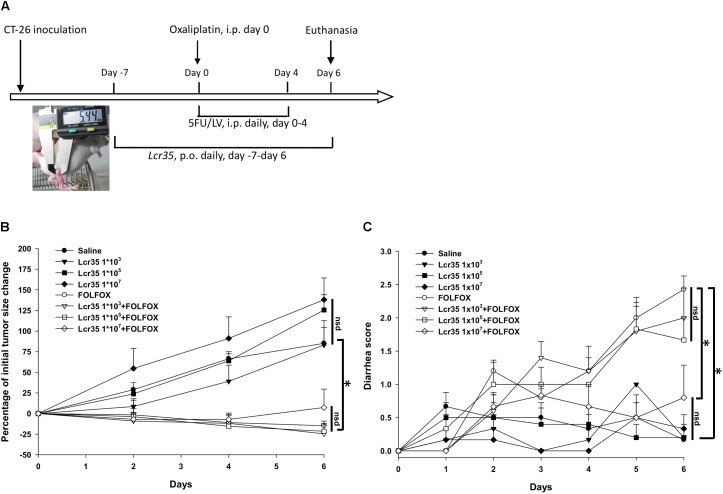
Effects of *Lcr35* in subcutaneously injected colorectal cancer mice challenged with FOLFOX (*n* = 6 for each group). **(A)** Protocols. **(B)** Antitumor activity in percentage. Tumor sizes are expressed as a percentage of that at day 0. **(C)** Diarrhea severity. Mice in each group were inoculated with CT26 cells (4 × 10^6^ cells) by subcutaneous injection into the right gluteal region. Treatment was started when the tumors reached 0.5 cm in diameter as measured with a caliper (insert in **A**); the mice were injected saline or FOLFOX i.p. daily for 5 days. Briefly, subcutaneously injected colorectal cancer mice in each control group and experimental group were orally administrated saline or probiotic suspension of *Lcr35* (1 × 10^3-7^ CFU/daily) 7 days before, during (days 0–4) and 2 days (days 5, 6) after FOLFOX administration i.p. for 14 days in total. Details of the experimental procedures are given in the Materials and Methods. ^∗^*p* < 0.05. nsd, no significant difference.

Male, 6- to 8-week-old BALB/c mice weighing 22–24 g were obtained from Taiwan’s National Laboratory Animal Center and were maintained under a 12-h light/dark cycle at a temperature of 22 ± 1°C and a humidity of 55 ± 10% (Yeung et al., 2015). Animal studies were performed in accordance with institutional ethical guidelines and approved by the Institutional Animal Care and Use Committee (IACUC) of MacKay Memorial Hospital (Taiwan) (MMH-A-S-104-15). All mice were given *ad libitum* access to autoclaved food (laboratory-autoclavable rodent diet 5010) and water. The mice were randomly divided into control and experimental groups. All mice were inoculated with CT26 cells (4 × 10^6^ cells) by subcutaneous injection into the right gluteal region. Treatment was started when the tumors reached 0.5 cm in diameter (insert in **Figure 1A**); the mice were injected saline or FOLFOX i.p. daily for 5 days (**Figure 1A**).

To evaluate the effect of probiotics, mice in the control and experimental groups were orally administered saline or a suspension of *Lcr35*, respectively, daily, 7 days before, during, and 2 days after FOLFOX administration, for a total of 14 days (**Figure 1A**, **Table 1**).

**Table 1:** Animal groups for evaluating the effects of *Lcr35* on FOLFOX-induced intestinal mucositis in subcutaneously injected colorectal cancer mice (*n* = 6 in each group).

Groups	Regimen
1	Saline
2	*Lcr35* (1 × 10^3^ CFU/day)
3	*Lcr35* (1 × 10^5^ CFU/day)
4	*Lcr35* (1 × 10^7^ CFU/day)
5	FOLFOX
6	*Lcr35* (1 × 10^3^ CFU/day) + FOLFOX
7	*Lcr35* (1 × 10^5^ CFU/day) + FOLFOX
8	*Lcr35* (1 × 10^7^ CFU/day) + FOLFOX

*Subcutaneously injected colorectal cancer mice in each group were orally administered saline or a suspension of Lcr35 (1 × 10^3-7^ CFU/daily) 7 days before, during (days 0–4), and 2 days (days 5, 6) after FOLFOX administration i.p. for a total of 14 days. Details of experimental procedures are provided in the Section “Materials and Methods.”*

Disease severity, including body weight, size of injected tumors, and diarrhea severity, was determined daily by a single observer. A caliper was used to measure the largest (a) and smallest (b) diameter, and the tumor volume was estimated according to the formula 0.5ab^2^ (Liu et al., 2005). Diarrhea severity was assessed by using Bowen’s score system (Bowen et al., 2007) and was classified into four grades according to the stool consistency: 0, normal stool; 1, slightly wet and soft stool, indicating mild diarrhea; 2, wet and unformed stool, indicating moderate diarrhea; and 3, watery stool, indicating severe diarrhea. Mice were euthanized 2 days after complete FOLFOX administration, and tissue and blood samples were harvested for histological and biochemical analyses (**Figure 1A**).

### Histological Analysis of Intestinal Injury

Intestinal tissues were removed and each harvested specimen was processed and fixed in 10% buffered neutral formalin for 2 h, dehydrated in an ascending series of ethanol concentrations, cleared in xylene, and embedded in paraffin wax. Sections of 4 μm thickness were cut and mounted on glass slides. Sections were routinely stained with hematoxylin and eosin (H&E) (Stringer et al., 2009b). Images were acquired using a 20× magnification objective. Specimens were viewed under a TissueFAXS automatic scanning system, captured by a digital camera, and analyzed with HistoQuest software (TissueGnostics) (Haisan et al., 2013). Villus height and crypt depth in the small intestine were measured for whole, well-oriented villi and crypts per small-intestinal tissue section per mouse, and the values were averaged.

### Immunohistochemical Analysis of Intestinal Injury

Immunohistochemistry was used to detect protein expression in the intestinal tissues. The sections were dewaxed with xylene and gradually hydrated. Heat-induced antigen retrieval was achieved by using 10 mM sodium citrate (pH 6) or EDTA (pH 8) buffer at 98°C for 15 min. Endogenous peroxidase activity was quenched with hydrogen peroxide for 10 min. Then, the sections were incubated in protein block solution for 10 min and rinsed with TBST. The sections were incubated with the primary antibodies at the indicated dilution at room temperature for 1 h or at 4°C overnight. As a negative control, a set of slides was processed without primary antibody. The following antibodies were used: anti-Ki-67 (ab16667; Abcam, 1:200 dilution); anti-CD44 (ab157107; Abcam, 1:2,000 dilution); anti-NF-κB (ab28856; Abcam, 1:50 dilution); anti-BAX (#14796; Cell Signaling Technology, 1:100 dilution); anti-BCL-2 (ab32124; Abcam, 1:100 dilution); and anti-caspase 8 (ab4052; Abcam, 1:50 dilution). The Polink-2 plus Polymer HRP Detection System (GBI Labs, Mukilteo) was used as the detection system. The 3,3-Diaminobenzidine was used as the chromogen and sections were counter-stained with hematoxylin. Apoptosis was quantified by terminal deoxyribonucleotide transferase (TdT)-mediated nick-end labeling (TUNEL) assay for detecting DNA breaks in the cells of various intestinal segments [*In Situ* Cell Death Detection Kit, POD (Roche)]. Goblet cells were stained with periodic acid–Schiff (PAS)/Alcian Blue (AB) stain (Alcian Blue-PAS Stain Kit; ScyTek Laboratories). Specimens were viewed under a TissueFAXS automatic scanning system, and images were captured with a digital camera and analyzed using HistoQuest software (TissueGnostics). Multiple (seven) images of intestinal tissue stained with PAS/AB, Ki67, CD44, TUNEL, NF-κB, BAX, BCL-2, and caspase-8 were acquired on high-power fields (200×) of four groups by using software for image analysis.

### Real-Time Quantitative (q)PCR

Total RNA from jejunum tissues was isolated using TRI Reagent^®^ RNA isolation Reagent (Sigma) according to the manufacturer’s instructions for animal tissue. Template cDNA was synthesized from RNA using reverse transcription with oligo(dT) (Stringer et al., 2009b) primers (Fermentas). DNA detection and amplification by qPCR was carried out on an ABI 7500 Sequence Detection System with system software version 1.2.3 (Applied Biosystems). Cytokines, including TNF-α, IL-1β, IL-6, and IL-10 were detected using the Maxima SYBR Green/ROX Q-PCR Master Mix (Applied Biosystems), with 100 nM of each of the forward and reverse primers and 1 ng of DNA per reaction. PCR cycles were as follows: 50°C for 2 min, 95°C for 10 min, and 40 cycles of 95°C for 15 s and 60°C for 1 min. Pairs of oligonucleotide primers specific to TNF-α, IL-1β, IL-6, IL-10, and the 18S rRNA housekeeping gene were used. The qPCR data were analyzed following the 2^-ΔΔ^*^C^*^t^ method using the 18S rRNA gene as an internal control. The relative quantity of the target transcript was described as a fold increase relative to the reference sample and 18S rRNA. Duplicate samples were routinely used for the determination of DNA by qPCR, and mean values were calculated.

### Gut Microbiota Analysis

#### DNA Extraction

Stool was sampled from mice (*n* = 4/group) and immediately stored at -80°C. DNA from fecal material was extracted with a QIAamp^®^ DNA Stool Mini Kit (Qiagen) according to the manufacturer’s instructions. The concentration was determined by using a NanoDrop 2000 Spectrophotometer (Thermo Scientific).

#### Sequence Analysis

The hypervariable region V3–V4 of bacterial 16S rRNA genes was amplified by PCR using bar-coded universal primers chosen according to (Nossa et al., 2010). Library construction and amplicon sequencing were conducted with Genomics BioScience. A pair-end library (insert size of 490 bp for each sample) was constructed with TruSeq Nano DNA Library Prep kit (Illumina) and high-throughput sequencing was carried out on an Illumina MiSeq sequencer with a MiSeq Reagent Kit v3 (Illumina). The sequences of 2 × 300-bp pair-end reads were produced from the sequencer following the manufacturer’s instructions. The pair-reads were merged into amplicon sequences using PEAR (Zhang et al., 2014) and these amplicon sequences were checked for the existence of the primers, duplicates were removed, and short sequences and chimeric reads were filtered out to generate effective reads. Effective reads were analyzed to generate operational taxonomic units (OTUs). Further 16S rDNA analysis (OTU picking and taxonomic assignment) and data visualization were conducted using Quantitative Insights Into Microbial Ecology (QIIME) version 1.8.0 (Caporaso et al., 2010) with the Greengenes 16S rRNA Taxonomy Database (gg_13_8). Taxonomy (i.e., phyla and OTUs) was analyzed by one-way ANOVA.

### *Lactobacillus* Growth Assay

#### Bacterial Strains, Growth Media, and Culture Conditions

*Lactobacillus casei* variety *rhamnosus* (*Lcr35*) (Antibiophilus^®^) was routinely activated and cultivated statically at 37°C for 24 h under aerobic conditions in MRS broth (BD 288130).

#### Determination of Minimum Inhibitory Concentrations (MICs)

Adjust *Lcr35* concentration to obtain an approximate final concentration of 3 × 10^5^ CFU/mL (Florez et al., 2016). The MICs of 5-fluorouracil (5-FU, Sigma F6627) and oxaliplatin (Sigma O9512) for *Lcr35* were determined using a broth dilution test for antibacterial testing as recommended by Clinical and Laboratory Standards Institute (CLSI) protocol (Balouiri et al., 2016). Aliquots of 100 μL of the diluted cell suspensions were added to MRS Agar plates (BD 288310), with the concentration of the 5-FU and oxaliplatin would be two-fold dilutions from 0 to 512 μg/mL. Absorbance was measured according to the CLSI guidelines (Balouiri et al., 2016). MICs were established as the lowest concentration of 5-FU and oxaliplatin at which no growth was observed. The experiments were performed in triplicate.

### Statistical Analysis

Results are presented as the mean ± standard error of the mean (SEM). Statistical significance (*p* < 0.05) was determined by one-way ANOVA. Data were analyzed with IBM SPSS software (version 21.0; SPSS Institute).

## Results

### Effects of *Lcr35* on Subcutaneously Injected Colorectal Cancer Mice Challenged With FOLFOX

Subcutaneously injected colorectal cancer mice were divided into eight treatment groups (**Table 1**), and experimental animals received dosage of *Lcr35* (1 × 10^5-7^ CFU/daily) orally 7 days before, during (days 0–4), and 2 days (days 5, 6) after FOLFOX treatment (**Figure 1A**). After completion of the experiment, no animal exhibited signs of marked adverse effects, such as bloody stool passage or cachexia. No mortality was noted.

Size of subcutaneously injected tumors was recorded. Tumor growth was significantly prevented in colon cancer-bearing mice challenged with FOLFOX as compared to saline controls (**Figure 1B**). At the end of the study (day 6), tumor size was significantly decreased in the FOLFOX group as compared to the saline control group (-17.75 ± 5.63% vs. 85.15 ± 19.17%; *p* < 0.005). *Lcr35* (1 × 10^3-7^ CFU/daily) alone did not affect tumor growth as compared to the saline control group (day 6; *Lcr35* 1 × 10^3^ vs. saline, *p* = 0.94; *Lcr35* 1 × 10^5^ vs. saline, *p* = 0.15; *Lcr35* 1 × 10^7^ vs. saline, *p* = 0.06) (**Figure 1B**). Furthermore, *Lcr35* (1 × 10^3-7^ CFU/daily) did not affect anti-tumorigenic activities of FOLFOX as compared to the FOLFOX group (day 6; *Lcr35* 1 × 10^3^ + FOLFOX vs. FOLFOX, *p* = 0.75; *Lcr35* 1 × 10^5^ + FOLFOX vs. FOLFOX, *p* = 0.81; *Lcr35* 1 × 10^7^ + FOLFOX vs. FOLFOX, *p* = 0.42).

Diarrhea scores were recorded daily, and the results of all groups were compared. In the four saline-treated groups (with or without probiotics), no significant diarrhea was detected (day 6; *Lcr35* 1 × 10^3^ vs. saline, *p* = 0.94; *Lcr35* 1 × 10^5^ vs. saline, *p* = 0.94; *Lcr35* 1 × 10^7^ vs. saline, *p* = 0.69), and their total scores remained < 1 throughout the experiment. However, after FOLFOX injection, the mice experienced the strongest diarrhea on day 6 (2 days after completion of FOLFOX treatment) as compared with saline group (2.7 ± 0.3 vs. 0.2 ± 0.2, *p* < 0.005; **Figure 1C**). On day 6, diarrhea severity was clearly attenuated in mice pretreated with the highest dose of *Lcr35* (1 × 10^7^ CFU/daily) in the FOLFOX group (0.8 ± 0.5 vs. 2.7 ± 0.3, *p* < 0.005; **Figure 1C**).

### Effects of *Lcr35* on FOLFOX-Induced Intestinal Mucosal Damage in Subcutaneously Injected Colorectal Carcinoma Mice

**FIGURE 2:**
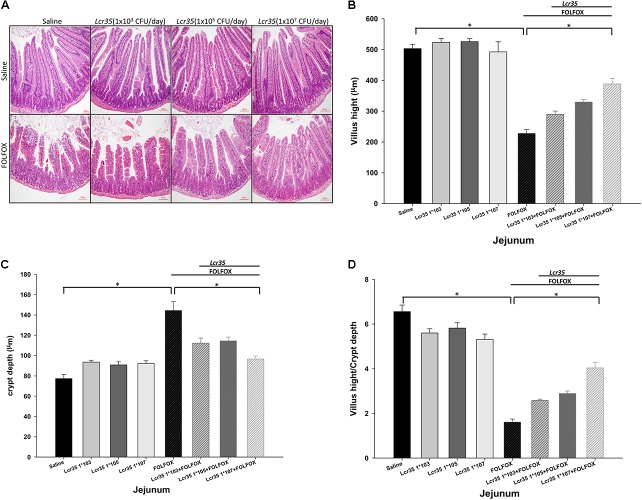
Effects of *Lcr35* on histological changes in the jejunum of subcutaneously injected colorectal carcinoma mice presenting FOLFOX-induced intestinal mucositis (*n* = 6 for each group). Segments of the jejunum were harvested for **(A)** hematoxylin and eosin staining (scale bar = 100 μm) and measurement of **(B)** villus height, **(C)** crypt depth, and **(D)** villus height-to-crypt depth ratio per mouse. The mice received saline or *Lcr35* (1 × 10^3-7^ CFU/daily) daily during the experiment. Values are presented as the mean ± SEM. ^∗^*p* < 0.05.

On day 6, FOLFOX caused substantial histological changes in the intestinal mucosal layer (**Figure 2A**), including flattened epithelial layer, shortened villi, and lamina propria with inflammatory cell infiltration in the intestine. The crypts were thickened.

FOLFOX significantly decreased villus height in the jejunum as compared to the control groups (**Figure 2B**). This effect was significantly and dose-dependently abrogated by *Lcr35* (1 × 10^5-7^ CFU/daily) in FOLFOX-injected mice, resulting in significantly lengthened villi as compared to the FOLFOX group (**Figure 2B**). Additionally, FOLFOX significantly increased intestinal crypt depth as compared to the control group (**Figure 2C**), while the crypt depth in the jejunum was significantly and dose-dependently restored by *Lcr35* (1 × 10^5-7^ CFU/daily; **Figure 2C**). Changes in villus height-to-crypt depth ratio were similar to those in villus height. FOLFOX markedly decreased the ratio in jejunal sections when compared to the control groups. These effects were also abrogated by *Lcr35* (1 × 10^5-7^ CFU/daily, *p* < 0.05; **Figure 2D**). *Lcr35* treatment alone did not markedly affect intestinal histology (**Figures 2A–D**). Therefore, oral administration of the probiotics *Lcr35* at the highest dose, 1 × 10^7^ CFU/daily, was the most effective in ameliorating FOLFOX-induced intestinal mucosal damage in colorectal carcinoma-bearing mice, as characterized by villus height (388.21 ± 17.83 vs. 227.46 ± 12.84 μm, *p* < 0.05), crypt depth (96.68 ± 2.79 vs. 144.39 ± 8.88 μm, *p* < 0.05), and villus height-to-crypt depth ratio (4.04 ± 0.24 vs. 1.61 ± 0.14, *p* < 0.05).

### Effects of *Lcr35* on Mucus Barrier Function of Villi and Proliferation, Regeneration, and Apoptosis of Crypt in Subcutaneously Injected Colorectal Cancer Mice After FOLFOX Treatment

**FIGURE 3:**
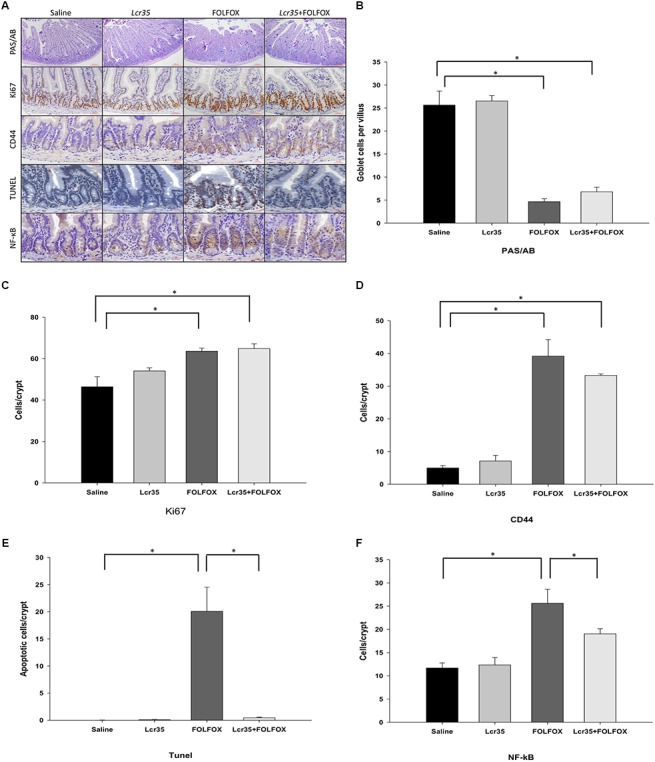
Effects of *Lcr35* on goblet cells, proliferation, regeneration, apoptosis, and NF-κB activity in FOLFOX-induced intestinal damage in subcutaneously injected colorectal carcinoma mice. **(A)** Immunohistochemical staining of jejunum sections was used to determine *Lcr35* effects on PAS (dark purple)/AB staining (goblet cells), Ki67 (brown) expression to detect proliferative activity, CD44 (brown) staining to detect regeneration activity, TUNEL (brown) to detect DNA breaks, and NF-κB activity (brown) in the intestine using antibodies. Quantification of the staining intensity of **(B)** PAS/AB in the intestinal villus, **(C)** Ki67, **(D)** CD44, **(E)** TUNEL, and **(F)** NF-κB in the intestinal crypt in **(A)**. Scale bar = 100 μm. The mice received saline or *Lcr35* (1 × 10^7^ CFU/daily) daily during the experiment. Results are representative of four individual experiments and are presented as the mean ± SEM. ^∗^*p* < 0.05.

Compared to the control group, PAS/AB staining showed a significant decrease in mucin-filled goblet cell number in the intestinal villus after FOLFOX injection (4.66 ± 0.64 vs. 25.63 ± 3.04 cells/crypt, *p* < 0.005). *Lcr35* at the highest dose, 1 × 10^7^ CFU/daily, did not significantly reduce goblet cell damage in the *Lcr35*/FOLFOX group, suggesting that *Lcr35* prevented villus damage without significantly affecting goblet cell differentiation and mucus barrier function in the intestine (**Figures 3A,B**).

Proliferative activity in intestinal crypts was estimated by crypt Ki67 expression in jejunal segments (**Figures 3A,C**). A total of 2 days after FOLFOX injection, the number of Ki67-positive cells was significantly increased in the FOLFOX group as compared to the saline group (63.57 ± 1.54 vs. 46.43 ± 4.85 cells/crypt, *p* < 0.01). *Lcr35* (1 × 10^7^ CFU/daily) administration did not affect the proliferative activity after FOLFOX injection in the *Lcr35*/FOLFOX group (**Figures 3A,C**).

To establish whether crypts in jejunal segments were repopulated by intestinal progenitor cells, we stained the cells with an antibody against CD44. Compared to the saline group, *Lcr35* (1 × 10^7^ CFU/daily) alone had no effect on the level of CD44. The number of CD44-positive cells at the bottom of the crypts significantly increased after FOLFOX or *Lcr35*/FOLFOX treatment (39.18 ± 5.06 or 33.28 ± 0.47 vs. 4.98 ± 0.77 cells/crypt, *p* < 0.05; **Figures 3A,D**). However, the number of CD44-positive cells was not different between the *Lcr35*/FOLFOX and FOLFOX groups (**Figures 3A,D**).

The TUNEL staining was performed to detect apoptotic cells in the intestinal tissues. FOLFOX administration caused a marked increase in TUNEL-positive apoptotic cells in the intestinal crypts. The number of apoptotic cells in the FOLFOX-treated group was about 20-fold that of the saline group (**Figures 3A,E**). *Lcr35* (1 × 10^7^ CFU/daily) significantly reduced FOLFOX-increased apoptotic cells (20.07 ± 4.47 vs. 0.45 ± 0.12 cells/crypt, *p* < 0.0001). *Lcr35* accelerated the disappearance of TUNEL-positive cells in the *Lcr35*/FOLFOX group across the crypts of jejunal segments (**Figures 3A,E**).

Immunohistochemical staining with an antibody against the NF-κB p65 subunit revealed that, compared to the saline group, there were many p65-reactive cells in the crypts of FOLFOX-treated intestinal tissues (**Figures 3A,F**). *Lcr35* (1 × 10^7^ CFU/daily) significantly reduced the FOLFOX-induced increase in the number of p65-reactive cells in the intestine (19.03 ± 1.12 vs. 25.60 ± 3.05 cells/crypt, *p* < 0.05). These findings indicated that FOLFOX-induced NF-κB activity, while *Lcr35* inhibited FOLFOX-induced NF-κB activity across the crypts of jejunal segments (**Figures 3A,F**).

### Effects of *Lcr35* on BAX, BCL-2, and Caspase-8 Expression in the Intestine of Subcutaneously Injected Colorectal Cancer Mice After FOLFOX Treatment

**FIGURE 4:**
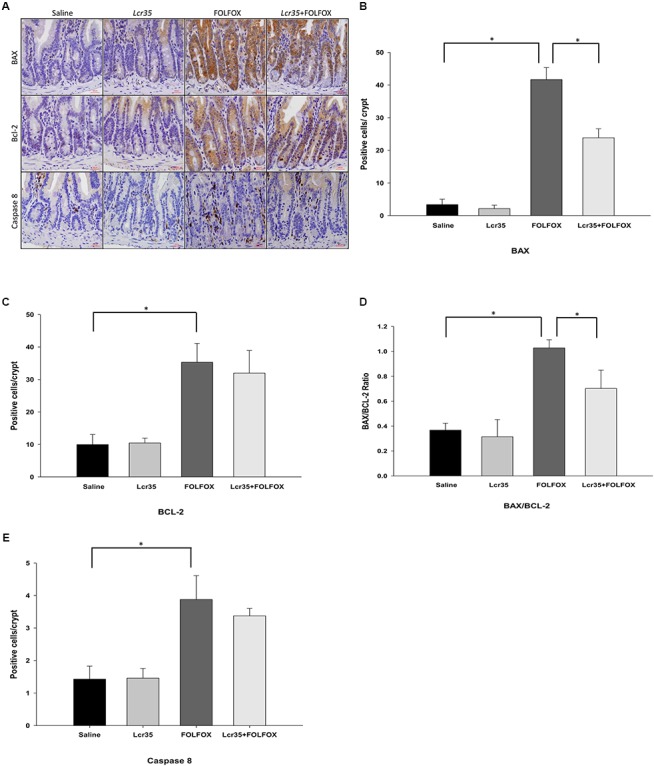
Effect of *Lcr35* on BAX, BCL-2, and caspase 8 expression in FOLFOX-induced intestinal damage in subcutaneously injected colorectal carcinoma mice. Immunohistochemical staining of jejunum sections and quantification of the staining intensity in the crypts of the intestine were conducted to determine *Lcr35* effects on **(A,B)** BAX, **(A,C)** BCL-2 activity (brown), **(D)** BAX/BCL-2 ratio, and **(A,E)** caspase 8 (brown) using antibodies. Scale bar = 100 μm. The mice received saline or *Lcr35* (1 × 10^7^ CFU/daily) daily during the experiment. Results are representative of four individual experiments and are presented as the mean ± SEM. ^∗^*p* < 0.05.

To elucidate the role of apoptotic cascades, the expression of caspase-8, BAX, and BCL-2 proteins was measured. To assess whether *Lcr35* had an effect on the expression and localization of mitochondria-associated apoptotic proteins, the numbers of BAX- and BCL-2-positive cells in the small intestines from mice in each group were quantified by immunohistochemical staining. Compared to the saline group, there were many BAX-positive cells in the crypts of FOLFOX-treated intestinal tissues (**Figures 4A,B**). *Lcr35* (1 × 10^7^ CFU/daily) significantly reduced the FOLFOX-induced increase in the number of BAX-positive cells in the intestinal crypts (23.83 ± 2.78 vs. 41.66 ± 3.69 cells/crypt, *p* < 0.05). These findings indicated that FOLFOX evoked BAX expression, while *Lcr35* inhibited FOLFOX-induced BAX expression in the intestine. Compared to the saline group, there were many BCL-2-positive cells in the crypts of FOLFOX-treated intestine (**Figures 4A,C**). *Lcr35* had no significant effect on the FOLFOX-induced increase in BCL-2 expression in the intestine. However, changes in the BAX/BCL-2 ratio were similar to that in BAX. FOLFOX markedly increased the ratio as compared to that observed in the saline control. These effects were reduced by *Lcr35* (1 × 10^7^ CFU/daily) administration in FOLFOX-injected mice (0.703 ± 0.06 vs. 1.03 ± 0.07 BAX/BCL-2 ratio, *p* < 0.05; **Figure 4D**).

FOLFOX administration caused a marked increase in the number of caspase 8-positive cells in the intestinal crypts as compared to the saline group. The *Lcr35* (1 × 10^7^ CFU/daily) administration had no effect on the FOLFOX-induced increase in caspase-8 protein expression across jejunal segments (**Figures 4A,E**).

### Effects of *Lcr35* on the Regulation of IL-1β, IL-6, TNF-α, and IL-10 mRNA Expression in the Jejunum of Subcutaneously Injected Colorectal Cancer Mice Challenged by FOLFOX

**FIGURE 5:**
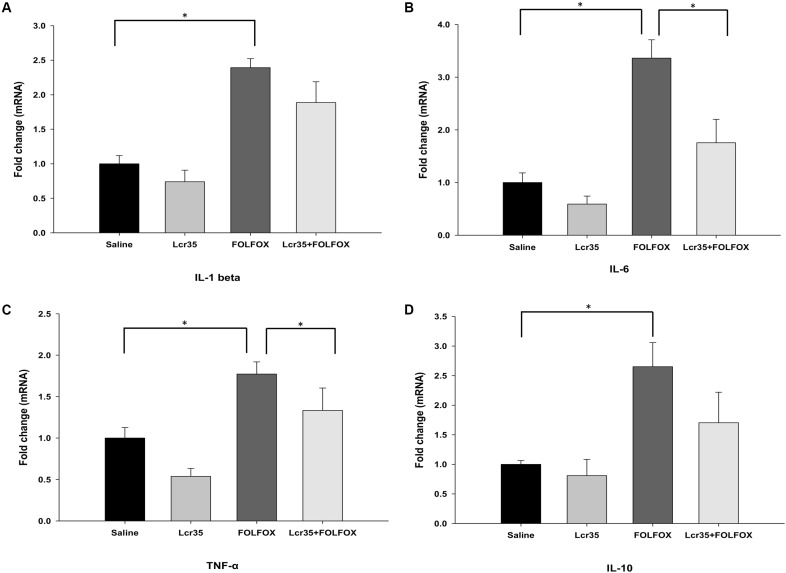
Effects of *Lcr35* on *Il-1β*, *Il-6*, *Tnf-α*, and *Il-10* mRNA expression in the jejunum of subcutaneously injected colorectal cancer mice challenged with FOLFOX. Gene expression of **(A)** IL-1β, **(B)** IL-6, **(C)** TNF-α, and **(D)** IL-10 was determined by Q-PCR in jejunum tissues. The mice received saline or *Lcr35* (1 × 10^7^ CFU/daily) daily during the experiment. Values are presented as the mean ± SEM. ^∗^*p* < 0.05.

After euthanasia, the effects of *Lcr35* (1 × 10^7^ CFU/daily) treatment on IL-1β, IL-6, TNF-α, and IL-10 mRNA expression in the jejunum of mice treated with FOLFOX were determined. IL-1β, IL-6, TNF-α, and IL-10 mRNA expression in the jejunum tissues was markedly upregulated in the FOLFOX-challenged group (**Figures 5A–D**). *Lcr35* (1 × 10^7^ CFU/daily) treatment significantly suppressed FOLFOX-induced IL-6 and TNF-α upregulation in jejunum tissues (1.76 ± 0.45 vs. 3.36 ± 0.35 and 1.12 ± 0.22 vs. 1.77 ± 0.15, respectively, *p* < 0.0.5; **Figures 5B,C**).

### Effects of *Lcr35* on Gut Microbiota Composition in Stool From Subcutaneously Injected Colorectal Cancer Mice Challenged With FOLFOX

**FIGURE 6:**
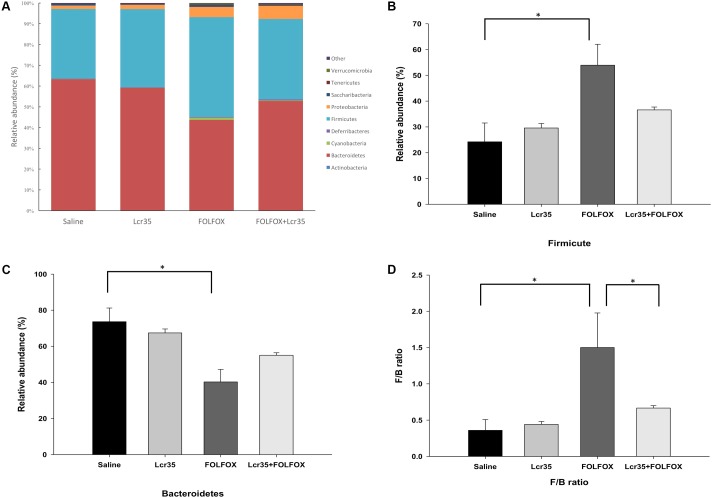
Effects of *Lcr35* on changes in the gut microbiota from stool of subcutaneously injected colorectal cancer mice challenged with FOLFOX. Gut microbiota composition was determined by **(A)** taxonomy at phylum level, **(B)** *Firmicutes*, **(C)** *Bacteroidetes*, and **(D)** *Firmicutes*-to-*Bacteroidetes* (F/B) ratio. The mice received saline or *Lcr35* (1 × 10^7^ CFU/daily) daily during the experiment. Values are presented as the mean ± SEM. ^∗^*p* < 0.05.

Fecal gut microbiota composition was determined by next-generation sequencing. Taxonomic analysis at the phylum level indicated that FOLFOX changed the gut microbiota composition. *Lcr35* altered this composition in the FOLFOX-challenged group as compared to the saline group (**Figure 6A**). *Bacteroidetes* and *Firmicutes* were the two major phyla in all groups. FOLFOX significantly increased the abundance of *Firmicutes* (F) as compared to the saline control (53.92 ± 8.11 vs. 24.23 ± 7.28 relative abundance (%), *p* < 0.05; **Figure 6B**). Moreover, FOLFOX significantly decreased the abundance of *Bacteroides* (B) as compared to the saline control [40.27 ± 6.94 vs. 73.64 ± 7.56 relative abundance (%), *p* < 0.05; **Figure 6C**]. Changes in the F/B ratio were similar to those in *Firmicutes* abundance. FOLFOX markedly increased the ratio as compared to the saline control. These effects were abrogated by *Lcr35* (1 × 10^7^ CFU/daily) administration in FOLFOX-injected mice (0.67 ± 0.03 vs. 1.50 ± 0.48 F/B ratio, *p* < 0.05; **Figure 6D**). *Lcr35*-treatment alone did not affect fecal gut microbiota composition. Thus, oral administration of probiotics, *Lcr35*, specifically altered FOLFOX-induced gut microbiota change in subcutaneously injected colorectal carcinoma mice.

### Effects of 5-FU and Oxaliplatin on *Lactobacillus* Growth

The MICs obtained for the *Lactobacillus casei* variety *rhamnosus* after 48 h of incubation. *Lactobacillus casei* variety *rhamnosus* grew at the highest concentrations of 5-FU and oxaliplatin (MICs > 512 μg/mL). *Lactobacillus casei* variety *rhamnosus* is resistant at the highest concentrations assayed [MICs > 512 μg/mL] to 5-FU and oxaliplatin *in vitro.* The 5-FU and oxaliplatin could not perturb the *Lactobacillus casei* variety *rhamnosus.*

## Discussion

To our knowledge, this is the first report demonstrating the potential of probiotics to suppress FOLFOX-induced mucositis in a colorectal cancer mouse model *in vivo*. Continuous FOLFOX administration to subcutaneously injected colon cancer mice significantly prevented tumor growth. The probiotic *Lcr35* dose-dependently ameliorated FOLFOX-induced severe diarrhea and intestinal mucosal injury in CT26 colorectal cancer-bearing mice. Furthermore, *Lcr35* reduced FOLFOX-induced intestinal mucosal inflammation characterized by immunohistological change and pro-inflammatory cytokine expression. FOLFOX-induced intestinal mucosal apoptosis and fecal gut microbiota changes were also mitigated by *Lcr35*. Together, these results indicated that *Lcr35* is clinically promising and relevant for the prevention or management of chemotherapy-induced mucositis.

Few studies have investigated gastrointestinal mucositis resulting from combined 5-FU and oxaliplatin chemotherapy in animal models and little is known regarding the pathophysiology of mucositis with this combination (Nukatsuka et al., 2012; Wang X. et al., 2015). Our study showed that marked diarrhea developed in the FOLFOX groups. Oral *Lcr35* administration significantly attenuated diarrhea and improved diarrhea scores. Furthermore, histological analysis of intestinal mucosal injury indicated that FOLFOX caused significant villus shortening in the mouse model. This damage in jejunal villi was dose-dependently prevented by *Lcr35* administration. Additionally, FOLFOX significantly lengthened the intestinal crypts, while *Lcr35* administration restored the crypt depth in FOLFOX-treated mice. Besides villus shortening, FOLFOX caused significant decreases in the villus height-to-crypt depth ratio in the tumor-bearing mouse model, which was alleviated dose-dependently by *Lcr35* administration in FOLFOX-injected mice, although the levels did not reach those observed in the normal saline group. Our results showing that FOLFOX-induced intestinal mucositis was consistent with that previously reported in a chemotherapy-induced intestinal mucositis mouse model (Justino et al., 2015; Wang X. et al., 2015; Yeung et al., 2015). However, the reported effects of probiotics on chemotherapy-induced mucositis on villus height and crypt depth are inconsistent (Prisciandaro et al., 2011b; Justino et al., 2015; Yeung et al., 2015; Tang et al., 2017), which might be owing to differences in probiotic strains, regimens, experimental protocols, or animal models used. Furthermore, these studies were mainly performed in healthy animal models. In contrast, we investigated chemotherapy-induced intestinal mucositis in tumor-bearing mice to mimic advanced colon cancer in humans.

The NF-κB is a transcription factor that regulates the expression of numerous genes that are critical for survival. NF-κB controls inflammation, cell growth, apoptosis, and cell cycle (Logan et al., 2007). It plays an important role in the pathobiology of mucositis. NF-κB activation induced by anti-neoplastic agents, such as 5-FU, is therefore thought to elicit inflammatory and apoptotic responses in the intestine (Logan et al., 2007; Chang et al., 2012). NF-κB activation results in the upregulation of the pro-inflammatory cytokines TNF, IL-1β, and IL-6 (Logan et al., 2007), leading to mucosal injury. Accordingly, inhibition of NF-κB has been suggested as an attractive strategy for the prevention of mucositis. In this study, immunohistological analysis indicated that NF-κB, induced by FOLFOX in the intestine, was the critical molecule regulating FOLFOX-induced intestinal mucositis. *Lcr35* administration decreased FOLFOX-induced NF-κB activity in the intestine and improved mucositis, as evidenced by changes in the histological characteristics. These findings suggest that inhibition of NF-κB activity by *Lcr35* might result in the suppression of inflammation and the sequential amelioration of mucositis in the intestine. Furthermore, different cytokines are responsible for the various stages of mucositis. Upregulation of pro-inflammatory cytokines, including TNF-α, IFN-γ, IL-1β, and IL-6, causes mucosal injury, eliciting further tissue damage (Sonis, 2004; Logan et al., 2007). We showed that TNF-α, IL-1β, and IL-6 expression was significantly upregulated in the jejunum of mice in the FOLFOX group, while probiotic administration abrogated this effect of TNF-α and IL-6. Thus, *Lcr35* attenuates the severity of intestinal mucositis induced by FOLFOX treatment in subcutaneously injected colorectal cancer mice through inhibition of the expression of pro-inflammatory cytokines involved in the pathogenesis of mucositis.

Apoptosis is a particularly critical event in intestinal mucositis induced by chemotherapeutic agents (Keefe et al., 2000; Bowen et al., 2006; Logan et al., 2007). Caspases and proteins from the BCL-2 family play an important role in apoptosis (Bowen et al., 2006). The activation of caspases 3, 8, and 9 plays a central role in early apoptosis, regulated by various factors, including the BCL-2 protein family (Bowen et al., 2006; Kumar, 2007). Activation of caspases in response to cancer chemotherapy can be initiated via two signaling pathways: the intrinsic and the extrinsic pathway (Fulda and Debatin, 2006). The intrinsic pathway is generally activated by DNA damage and when pro-apoptotic proteins such as BAX are released from mitochondria. This mitochondria-mediated apoptotic pathway, regulated by members of the BCL-2 family, depends on the balance of the anti-apoptotic protein, BCL-2, and the pro-apoptotic protein, BAX (Breckenridge and Xue, 2004). Several studies have demonstrated that 5-FU-induced apoptosis is accompanied by an increase in pro-apoptotic BAX expression and a decrease in anti-apoptotic BCL-2 expression through the intrinsic apoptotic pathway (Inomata et al., 2002; Wu et al., 2011). In contrast, the extrinsic pathway is initiated extracellularly via activation of death receptor signaling by factors such as TNF-α. Caspase-8 is an essential component of the extrinsic cell death pathway initiated by the TNF family members (Kumar, 2007). Caspase-3, the main downstream effector caspase, is activated following cleavage by caspase-8 or -9 (Kumar, 2007). In the present study, the number of TUNEL-positive apoptotic cells markedly increased in the intestinal crypts after FOLFOX administration. *Lcr35* significantly reduced FOLFOX-induced apoptosis of the intestinal crypt cells, suggesting that *Lcr35* ameliorates intestinal mucositis by suppressing apoptosis induced by FOLFOX. Furthermore, a remarkable increase in the BAX/BCL-2 ratio in the intestinal crypt cells of the FOLFOX group and a shift of the equilibrium of BCL-2 family members toward apoptosis were observed (Bowen et al., 2006). *Lcr35* reduced the FOLFOX-induced increase in the BAX/BCL-2 ratio and induced a shift toward anti-apoptosis. Additionally, caspase-8 was activated in the intestinal crypt cells of the FOLFOX group; however, *Lcr35* administration did not significantly reduce this activity. Thus, in intestinal mucositis, apoptosis in response to FOLFOX can be induced via both the extrinsic and intrinsic pathways, and *Lcr35* predominantly reduced FOLFOX-induced apoptosis via the intrinsic pathway.

Several local and systemic inflammatory diseases are linked to gut microbiota, including inflammatory bowel disease, radiotherapy-induced diarrhea, obesity, and diabetes (van Vliet et al., 2010; Boulange et al., 2016). Chemotherapy is associated with a change in microbial diversity (Stringer et al., 2009a,b; van Vliet et al., 2010; Touchefeu et al., 2014). This change in microbial diversity coincides with the development of severe chemotherapy-induced mucositis (van Vliet et al., 2010). Commensal intestinal bacteria could be incorporated as a meaningful factor in the Sonic’s five-phase model of the pathogenesis of mucositis (van Vliet et al., 2010). Accordingly, manipulating gut microbiota might have therapeutic potential against chemotherapy-induced mucositis. In the current study, next-generation sequencing revealed that FOLFOX changed the gut microbiota composition, and oral administration of *Lcr35* altered this compositional change. Further taxonomic analysis at the phylum level indicated that FOLFOX significantly increased the abundance of *Firmicutes*, decreased the abundance of *Bacteroidetes*, and increased the F/B ratio. These changes were restored by *Lcr35* administration. *Bacteroidetes* and *Firmicutes* are the predominant phyla in humans and mice (Eckburg et al., 2005; Touchefeu et al., 2014). The balance between these two phyla (F/B ratio) appears to be critical for the regulation of radiotherapy or chemotherapy-related mucositis (Touchefeu et al., 2014; Wang A. et al., 2015). Additionally, gut microbiota can interact with the extracellularly located parts of Toll-like receptors (TLRs) and activate the NF-κB signaling pathway, which triggers the production of proinflammatory cytokines, resulting in the development of an inflammatory response (van Vliet et al., 2010; Touchefeu et al., 2014). Our data suggest that *Lcr35* altered FOLFOX-induced gut microbiota change and influenced the pathogenesis of mucositis via the gut microbiota-TLRs-NF-κB signaling pathway in subcutaneously injected colorectal carcinoma mice.

The exact mechanisms by which probiotics exert their beneficial effects remain unknown. Potential mechanisms may include the inhibition of inflammatory pathways, maintenance of intestinal permeability and mucin secretion, prevention of cell apoptosis and oxidative damage, and prevention of pathogenic colonization by re-establishing the intestinal microflora (Prisciandaro et al., 2011a). Different probiotics demonstrate various beneficial effects. Moreover, not all studies have demonstrated beneficial effects of probiotics on chemotherapy-induced mucositis. This could be explained by the use of different probiotic strains and anti-neoplastic agents, and single or combination of probiotic strains in different mucositis animal models (Prisciandaro et al., 2011a). Prisciandaro et al. (2011a) have proposed that a combination of several probiotic strains may be more reliable and efficacious. In this study, we used a single strain, which mitigated chemotherapy-induced mucositis dose-dependently. NF-κB activated by FOLFOX may result in apoptotic signals and pro-inflammatory cytokine production in normal mucosal tissue and subsequently contribute to gastrointestinal injury. Probiotics (such as *Lcr35*) could modulate the gut flora composition, and inhibit inflammation and apoptosis.

Interestingly, our findings also indicate spontaneous regulation toward mucosa healing 2 days after FOLFOX cessation in this tumor mouse model (Logan et al., 2007). This negative feedback of mucositis included mucosal crypt proliferation, regeneration, and anti-apoptosis, assessed by Ki67, CD44, and BAX IHC staining, respectively. Additionally, the anti-inflammatory cytokine, IL-10, was also expressed (Sultani et al., 2012). *Lcr35* administration had no significant effect on this negative feedback. Autophagy is essential for cell survival and differentiation. Recent evidence indicates that the intestinal epithelium and autophagy act in concert to maintain gut homeostasis and the intestinal epithelium can induce a protective autophagy (Baxt and Xavier, 2015). In our study, autophagy was not detected in mice after FOLFOX or *Lcr35* administration (data not shown).

A number of studies have reported the benefits of probiotics against colorectal cancer (CRC) onset, mainly through participating in the modulation of the immune response and induction of cell apoptosis, antioxidant activity, and improving the community of gut microbiota (Ambalam et al., 2016; Meng et al., 2018). *In vivo* studies with chemical-induced animal models (1,2-dimethylhydrazine, DMH; 2,4,6-trinitrobenzenesulfonic acid, TNBS; azoxymethane, AOM, dextran sodium sulfate, DSS; MNNG) and knockout models have provided evidence that the administration of probiotics strains significantly protects against CRC (Ambalam et al., 2016; Meng et al., 2018). As a specific example, the probiotic strain *L. casei* BL23 displayed anti-tumor properties in a mouse allograft model of human papilloma virus-induced cancer and in a DMH-induced CRC model (Lenoir et al., 2016). Further, this strain prevented colitis-associated CRC development induced by AOM-DSS (Jacouton et al., 2017). *Lactobacillus* strains, *L. plantarum* and *L. acidophilus*, but not *L. rhamnosus* suppressed tumor growth in CT26 tumor-bearing mice (Chen et al., 2012; Hu et al., 2015). Our study revealed that *Lcr35* alone showed no benefits in terms of suppression of CRC and did not interfere the anti-tumor effect of FOLFOX in CT26 subcutaneous injection mice. These different anti-tumor activities of probiotics might be owing to differences in probiotic strains, regimens, experimental protocols, or animal models used. Furthermore, the previous studies were mainly performed in spontaneous and chemically induced CRC in rodents and had the advantage of investigating the effects of the probiotics on the suppression of CRC development in the colorectal region (Evans et al., 2016). In contrast, our study was mainly to investigate the protective effect of probiotics on chemotherapy-induced gastrointestinal toxicity (side effect), including diarrhea and intestinal mucositis. The animal model used in our study was established to mimic patients with colon cancer suffering from chemotherapy-associated gastrointestinal toxicity and diarrhea.

To clarify the effect of 5-FU and oxaliplatin on the growth of *Lcr35*, we have determined minimum inhibitory concentrations (MICs) to evaluate the inhibitory role of 5-FU and oxaliplatin *in vitro*. In our study, *Lactobacillus casei* variety *rhamnosus* grew at the highest concentrations of 5-FU and oxaliplatin far higher than those reached in plasma during anticancer treatment (Bocci et al., 2000; Graham et al., 2000; Florez et al., 2016). Florez et al. (2016) also demonstrated that most species of Lactobacilli, including *Lactobacillus casei* and *Lactobacillus rhamnosus*, were resistant to the highest concentrations of 5-FU assayed (MICs > 128 μg/mL) *in vitro* (Florez et al., 2016). Accordingly, 5-fluorouracil and oxaliplatin do not inhibit *Lactobacillus* growth *in vitro.* To get a complete picture of the impact of 5-FU and oxaliplatin on the *Lactobacillus* growth, further research *in vivo* is needed.

This study has several limitations. First, analysis of the time-course and dose-dependent effects of probiotics will provide more information regarding their role in the pathogenesis of chemotherapy-induced mucositis. Second, further *in vitro* studies for elucidating anti-mucositis mechanism are required. Third, our analyses did not identify specific microorganisms that are related to chemotherapeutic injuries. We only demonstrated that quantitative alterations in the proportions of several phyla, such as Firmicutes and Bacteroidetes, might be useful biomarkers of chemotherapy exposure in the gut. Future studies should focus on the functional roles and taxonomic analysis of chemotherapy-related microorganisms in the gastrointestinal tract. Concerning the impact of tumor microenvironment, the orthotopic or carcinogen-induced CRC models is worthy to be applied for further validation (Terracina et al., 2015; Evans et al., 2016). Clinical studies are also required to demonstrate the beneficial effects of probiotics and to elucidate the safety and correct regimens for the management of chemotherapy-induced mucositis.

## Conclusion

Our colorectal cancer murine model with intestinal mucositis induced by FOLFOX may be an effective model to investigate the mechanisms underlying intestinal injury and its possible interaction with drugs. The development of FOLFOX-induced mucositis involves changes in gut microbiota and might be “driven” by the activation of NF-κB. Activated NF-κB results in apoptotic signals and pro-inflammatory cytokine production, sequentially contributing to gastrointestinal injury. By modulation of the gut microbiota and proinflammatory responses with suppression of intrinsic apoptosis, *Lcr35* mitigated FOLFOX-induced mucositis and may serve as an alternative therapeutic strategy for the prevention or management of chemotherapy-induced mucositis in future.

## Author Contributions

C-WC, C-YL, T-HT, and Y-JC performed the study concept and design. C-WC, L-HL, and J-SCC performed the acquisition of data. C-WC, C-YL, L-HL, Y-HH, T-HT, and Y-JC performed the analysis and interpretation of data. H-CL, T-EW, C-HC, S-CS, T-HT, and Y-JC performed the critical revision of the manuscript for important intellectual content. C-WC, T-HT, and Y-JC drafted of the manuscript.

## Conflict of Interest Statement

The authors declare that the research was conducted in the absence of any commercial or financial relationships that could be construed as a potential conflict of interest.
